# Modeling behavioral thermoregulation in a climate change sentinel

**DOI:** 10.1002/ece3.1848

**Published:** 2015-11-24

**Authors:** Lucas Moyer‐Horner, Paul D. Mathewson, Gavin M. Jones, Michael R. Kearney, Warren P. Porter

**Affiliations:** ^1^ Department of BiologyUniversity of UtahSalt Lake CityUtah; ^2^ Department of ZoologyUniversity of Wisconsin‐MadisonMadisonWisconsin; ^3^ Department of Forest & Wildlife EcologyUniversity of Wisconsin‐MadisonMadisonWisconsin; ^4^ Department of ZoologyThe University of MelbourneMelbourneVictoriaAustralia

**Keywords:** Activity, American pika, biophysical model, elevation, Glacier National Park, Niche Mapper, *Ochotona princeps*, protected area, temperature

## Abstract

When possible, many species will shift in elevation or latitude in response to rising temperatures. However, before such shifts occur, individuals will first tolerate environmental change and then modify their behavior to maintain heat balance. Behavioral thermoregulation allows animals a range of climatic tolerances and makes predicting geographic responses under future warming scenarios challenging. Because behavioral modification may reduce an individual's fecundity by, for example, limiting foraging time and thus caloric intake, we must consider the range of behavioral options available for thermoregulation to accurately predict climate change impacts on individual species. To date, few studies have identified mechanistic links between an organism's daily activities and the need to thermoregulate. We used a biophysical model, Niche Mapper, to mechanistically model microclimate conditions and thermoregulatory behavior for a temperature‐sensitive mammal, the American pika (*Ochotona princeps*). Niche Mapper accurately simulated microclimate conditions, as well as empirical metabolic chamber data for a range of fur properties, animal sizes, and environmental parameters. Niche Mapper predicted pikas would be behaviorally constrained because of the need to thermoregulate during the hottest times of the day. We also showed that pikas at low elevations could receive energetic benefits by being smaller in size and maintaining summer pelage during longer stretches of the active season under a future warming scenario. We observed pika behavior for 288 h in Glacier National Park, Montana, and thermally characterized their rocky, montane environment. We found that pikas were most active when temperatures were cooler, and at sites characterized by high elevations and north‐facing slopes. Pikas became significantly less active across a suite of behaviors in the field when temperatures surpassed 20°C, which supported a metabolic threshold predicted by Niche Mapper. In general, mechanistic predictions and empirical observations were congruent. This research is unique in providing both an empirical and mechanistic description of the effects of temperature on a mammalian sentinel of climate change, the American pika. Our results suggest that previously underinvestigated characteristics, specifically fur properties and body size, may play critical roles in pika populations' response to climate change. We also demonstrate the potential importance of considering behavioral thermoregulation and microclimate variability when predicting animal responses to climate change.

## Introduction

Species respond to rising temperatures in numerous ways. For example, some shift the elevation or latitude of their range (Thomas 2010; Chen et al. 2011). However, before shifting geographically, individuals attempt to tolerate novel environmental conditions and maintain their mass/energy balance through behavioral thermoregulation (Huey et al. 2012). These earliest responses to environmental change may not initially reduce body condition below thresholds where individual survival or fecundity is impacted. However, as environmental conditions deviate further from the range of conditions to which organisms are ecologically adapted, individuals may be forced to make behavioral trade‐offs to maintain energy balance (Speakman and Król 2010). Ultimately, these behavioral changes may impact individual fecundity (Parker et al. 2009), which, on relatively short timescales, may lead to population‐level effects such as range shifts, local extirpations, and ultimately extinction (e.g., Sinervo et al. 2010).

To understand and predict the earliest behavioral responses to climate change, we must consider the biophysical mechanisms that limit the activity of organisms. Optimality theory states that organisms evolve to make behavioral decisions so as to maximize their fitness, and these decisions are informed by environmental cues that may have historically limited fitness (Westneat and Fox 2010). For example, at the height of a temperate growing season, organisms may limit foraging time when temperatures are high, and instead rest or take refuge in cooler microclimates. Although foraging confers essential fitness benefits through the improvement or maintenance of body condition, resting or taking refuge when temperatures are high may confer even greater fitness benefits by reducing heat gain and avoiding hyperthermia (Ricklefs and Miller 2000). These critical decisions are fundamentally governed by the biophysical parameters of organisms (i.e., metabolic rate, surface properties) in combination with the external environmental conditions (i.e., landscape, microclimate) in which the organism resides, which together comprise the underpinning features of biophysical animal models. Biophysical models may prove useful in predicting how different animal populations are affected by climate and how they may respond; essential information for management and recovery of imperiled species. For example, biophysical models may allow us to better prioritize conservation efforts by identifying species with the least ability to tolerate rising temperatures through behavioral thermoregulation and also species whose fundamental and realized niches may move beyond the borders of current protected areas (Monzon et al. 2011).

**Figure 1:**
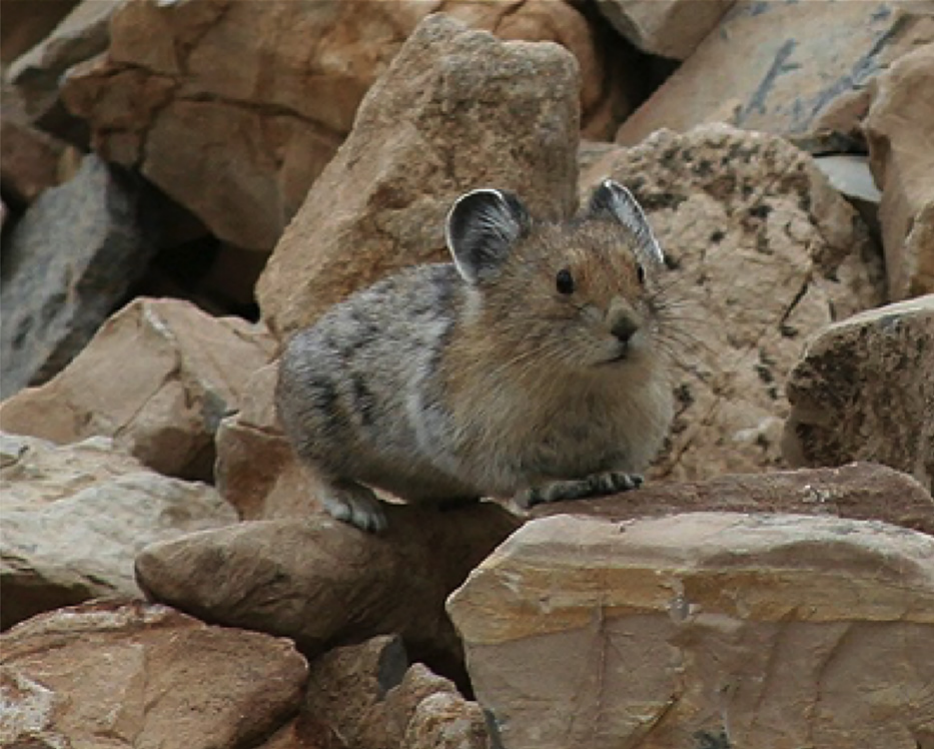
American pika, *Ochotona princeps,* in Glacier National Park, MT, USA. Photo by Wendee Nicole Holtcamp.

For many such species presumed and demonstrated to be at risk from climate change, considerable gaps in our understanding exist concerning the driving biological mechanisms, because fine‐scale data on the physiological effects of the thermal environment are rarely available (Beever and Belant 2011). Biophysical models therefore hold enormous potential for complementing traditional correlative approaches to fill gaps in knowledge for species of conservation concern. For the American pika (*Ochotona princeps,* Fig. 1), a temperature‐sensitive mammal inhabiting mountainous regions of the western Unites States (Smith and Weston 1990), such knowledge gaps were cited in a recent rejection of a petition to list the species as threatened by climate change under the Endangered Species Act (Crist 2010). In particular, more data are needed concerning pikas' abilities to tolerate high ambient temperatures through behavioral thermoregulation. This need, along with its known temperature sensitivity, makes the pika an ideal model organism for investigating temperature‐driven behavior modification from a biophysical perspective.

Pika basal metabolic rate (BMR) is high (143% of predicted weight‐specific value) and thermal conductance is low (101–53% of predicted values; MacArthur and Wang 1973), resulting in elevated and stable body temperatures (mean = 40.1°C) and relatively low upper‐lethal temperatures (mean = 43.1°C; Smith 1974). These factors suggest that behavior plays an important role in pika thermoregulation (MacArthur and Wang 1974). Indeed, hyperthermia and death may occur at moderate (25.5–29.4°C) ambient temperatures (MacArthur and Wang 1973; Smith 1974). These physiological sensitivities scale up to drive biogeographic distributional patterns in pikas; at high latitudes, *O. princeps* is found at elevations ranging from sea level to 4000 m, but pikas are rarely found below 2500 m where they meet their southern range boundary (Smith and Weston 1990). At these scales, pikas have responded to changes in climate both recently and historically by shifting upslope or becoming extirpated at low‐elevation sites across their range, suggesting vulnerability to rapid climate change in the 21st century (Hafner 1993; Grayson 2005; Moritz et al. 2008; Beever et al. 2010, 2011; Wilkening et al. 2011). However, pika declines are not range‐wide (e.g., Millar and Westfall 2010; Erb et al. 2011; Smith and Nagy 2015) and they persist at relatively warm and low‐elevation sites, especially where cooler and wetter microclimates exist (e.g., Beever 2002; Beever et al. 2008; Simpson 2009; Rodhouse et al. 2010; Jeffress et al. 2013; Varner and Dearing 2014). The mechanisms driving pika range shifts and extirpations in certain subpopulations but not others remain unclear, primarily because we do not know enough about their biophysical tolerances and capacity to behaviorally thermoregulate.

We present an analysis of environmental drivers of pika activity using a multifaceted approach that includes both the use of biophysical modeling and direct observations of pika activity. To do this, we (1) used temperature and microclimate data from the pika's thermal environment coupled with fur properties, body size, and wind speed to build a biophysical model (Niche Mapper, Porter and Mitchell 2006) that could predict metabolic rate and activity hours of the American pika. We subsequently tested the biophysical model by simulating empirical metabolic chamber data. Next, we (2) performed statistical analyses using 288 h of field observations of pika activity from a series of field sites in Glacier National Park (GNP), Montana, USA, to understand how environmental variables were associated with pika activity in the field. We also predicted the available activity time at a series of field sites using our biophysical model and qualitatively compared predicted values to observed pika activity at those sites. Finally, (3) we used the biophysical model to predict how pikas may be forced to respond behaviorally to projected 21st century climate change.

## Material and methods

### Building and validating the biophysical model

#### Microclimate data

We deployed AQA 8000 Series LogTag^™^ data loggers (www.logtagrecorders.com) at sites occupied by pikas in two stages. In the first stage, we used the loggers to estimate an altitudinal lapse rate for interpolating the elevation–temperature relationship across GNP. In June and July 2008, we placed seven loggers in protected crevices 1–2 m below the talus surface adjacent to a pika's food cache (or “hay‐pile”), one at each of seven sites with varying slope aspects and elevations ranging from 1512 to 2274 m. Two of these loggers at intermediate elevations were placed within 100 m of each other to assess small‐scale and potential logger variability. The loggers recorded temperature and relative humidity at 15‐min intervals. We calculated the lapse rate by fitting a linear regression to the instantaneous morning (07:00), midday (14:00), and evening (20:00) temperatures at each of the sites at one‐week intervals from July 28 to September 15.

In the second stage, we used four loggers to measure the thermal refuge provided by the talus by measuring above‐ and below‐talus temperature at two locations representing elevation extremes (1056 m and 2521 m). At both sites, one logger was placed, unshaded, on top of the talus. The other was placed under the same rock and next to a hay‐pile. These loggers recorded temperature and relative humidity at 20‐min intervals. We did not deploy loggers at all behavioral observation sites, so in order to interpolate above‐ and below‐talus temperatures at those sites, we applied our estimated lapse rate of −8°C/km (from stage one) to the stage‐two thermal refuge data. Our estimated lapse rate fell within the range considered appropriate for GNP (Ray et al. 2010).

#### Pika physical parameters

The pika physical properties used for biophysical modeling (see below) were either measured directly using a preserved pika specimen from the University of Wisconsin‐Madison Zoology Museum (with summer pelage, obtained in Colorado) or gleaned from the literature. We directly measured fur length and reflectivity for dorsal and ventral sections of the torso and legs. We assumed that winter fur was 50% longer than summer fur (Howell 1924), and that fur depth was always 50% of fur length. Animal model inputs are summarized in Table S1 of the Supporting Information.

#### Mechanistic model simulations

Niche Mapper is described in detail elsewhere (Porter and Mitchell 2006; Mathewson and Porter 2013). In brief, Niche Mapper consists of two submodels: a microclimate model and an endotherm model. The microclimate model computes the hourly values of local air temperature at animal height, ground surface temperatures, below ground soil temperatures, local wind speed and relative humidity, and direct and diffuse solar radiation in the sun and shade. The endotherm model numerically solves for metabolic rates that allow an animal to maintain its core temperature within a defined range under the computed environmental conditions. To do so, both models solve the generic heat balance model, *Q*_in_ *+ Q*_gen_ *= Q*_out_ *+ Q*_st_; where heat in (*Q*_in_) comes from solar and infrared radiation; *Q*_gen_ is metabolic heat generated (set to zero for the microclimate model); heat out (*Q*_out_), is infrared radiation from the surface (ground or fur–air interface), convective heat loss, and evaporative heat loss; and stored heat (*Q*_st_), which is set to zero in the endotherm model for steady‐state simulations. Niche Mapper solves these equations for each hour of the day, enabling analyses such as how many hours of a month's “average” day a model animal will be either heat or cold stressed when trying to maintain a given activity level (quantified as a multiple of BMR). If the metabolic rate that solves the heat balance equation is below the target activity level, the animal is predicted to overheat if active in those conditions and thus activity is not possible.

We tested Niche Mapper's ability to predict metabolic rates as a function of ambient air temperature prior to field simulations of pikas in GNP by simulating the metabolic chamber experiments of MacArthur and Wang (1973), using the same mean pika mass (109 g) and constant wind speed (0.1 m/s) used in the experiments. We also conducted a series of sensitivity analyses to determine the influence of various animal and environmental properties on the model's metabolic rate predictions.

### Field observations of pika activity in GNP

We observed pika activity at eight sites in GNP from 15 to 24 July, 2009. We chose sites that had relatively high pika densities across a broad range of elevations and slope aspects. We conducted twelve separate 3‐h observations per site during the following intervals: morning (05:15–08:15), midday (13:00–16:00), and evening (19:00–22:00). We conducted observations over a 10‐day span to reduce the effect of seasonality; pikas are more active in late summer when caching food (Smith and Weston 1990). Morning observations commenced ~45 min prior to sunrise and evening observations concluded ~30 min after sunset. We observed pika activity passively from a high vantage point, because pikas pay little attention to quiet observers (Conner 1983). Trained observers continuously scanned the talus using the naked eye or 7 × 35 binoculars, and recorded the time of all visual and audible evidence of pika activity. Observers worked in pairs, but recorded data from nonoverlapping areas (~40 m radius) of the same talus patch, assigning pikas temporary ID numbers and sketching maps of their whereabouts to distinguish the activity of individuals (sensu Barash 1973). A single pika call or sighting was considered a “unique event”. Additional calls or sightings of the same individual within 30 sec were recorded, but not considered new unique events. In this way, we categorized brief flourishes of activity as single events. We identified the type of observed behavior under the following nonmutually exclusive categories: haying (collecting or carrying vegetation in the mouth), foraging (directly consuming vegetation), moving fast, moving slow, and vigilant (stationary). We assumed that imperfect detection of pikas played a relatively small role in data collection, because pikas are highly detectable (85–95%) and relatively easy to identify for a stationary observer (Beever et al. 2010; Moyer‐Horner et al. 2012).

We used multiple regression to associate minimum above‐talus temperature (*t*) and two site‐level variables (elevation, *e*; slope aspect, *a*) with aggregate above‐talus pika activity observations to understand whether field observations were generally congruent with predictions from our biophysical model. We only modeled pika activity with minimum above‐talus temperature because of moderate to high collinearity with other temperature measurements (0.55 < *r *<* *0.99) and because minimum above‐talus temperature best explained variability in pika activity in preliminary analysis (see Table S2). There was no strong collinearity detected among the remaining predictor variables (*r *=* *0.06–0.26). We developed a set of 10 a priori hypotheses relating minimum above‐talus temperature, elevation, and slope aspect to observed pika activity (see Table S3) and evaluated relative empirical support for these hypotheses using AIC_C_ (Burnham and Anderson 2002). We derived parameter estimates that accounted for model selection uncertainty by performing model averaging among models with >10% AIC weight and without uninformative parameters (Burnham and Anderson 2002; Arnold 2010). We log‐transformed the response variable (per capita pika sightings) to approximate a normal distribution (Ives 2015). Log‐linear forms of *e*,* a*, and *t* outperformed linear and quadratic forms in preliminary analysis (see Fig. S1); therefore, we used log‐linear forms of our predictor variables in the final analysis. We fit models and performed model selection using the package ‘AICcmodavg’ (Mazerolle 2013) in R version 3.1.1 (R Core Development Team).

### Field simulations

Niche Mapper field simulations were conducted using a diurnally active, ellipsoidal (2.25 a:b ratio) pika and a wind velocity at pika height (9 cm) varying diurnally from 0.1 to 4.0 m/s. Hair lengths and depths were scaled according to the total mass of the modeled animal, for example, a 120 g pika had 20% shorter and shallower hair than a 150 g pika. Climate and weather data were retrieved from the National Oceanic and Atmospheric Administration website (www.noaa.gov) for Kalispell International Airport ([KIA] elevation 961 m), which is located ~30 km from Glacier National Park. When appropriate, we used a lapse rate of −8°C/km to derive mean monthly maximum and minimum temperatures (see below). In addition, we conducted sensitivity analyses to test Niche Mapper's robustness to input variable uncertainty (see Tables S1 and S4) and also compared its calculated above‐ and below‐talus temperatures to those we measured in the field. For simulations used as direct comparisons to field observations, we used site‐specific parameters, such as aspects and elevations of those sites, along with adjusted KIA temperatures from those days. For all other simulations, we used an eastern aspect, a 15° slope, GNP‐relevant elevations of 1500–2500 m, and long‐term (1981–2010) monthly averages from KIA.

We simulated a conservative warming scenario for 2100 of +5.0°C for all elevations (McWethy et al. 2010). We used daily temperature ranges of 20°C, typical for summer months in GNP. The lowest temperature range (−10–10°C) is typical for June or September above 2500 m elevation. July and August temperature ranges at the lowest elevations in GNP (1500 m) are typically 4–22°C. We interpreted the ratio of the field metabolic rate over the BMR (FMR/BMR) to determine when pikas could be active above the talus. If an FMR/BMR ratio below 1.5 was required to remain below the animal's upper critical temperature, activity would probably be infeasible (Nagy 1987).

## Results

### Building and validating the biophysical model

#### Microclimate data

The observed altitudinal lapse rate varied depending on the prevailing daily weather patterns and time of day. Temperature inversions were more frequent during mornings (07:00) and in September (see Tables S5–7). We measured a consistent lapse rate (*R* ^2 ^> 0.75) during seven of 24 measurements, for which the mean lapse rate was −9.46°C/km. The midday (14:00) mean for July and August measurements was −8.08°C/km.

The difference between below‐ and above‐talus temperature varied considerably depending on the time of day, and generally below‐talus temperatures were less variable than above‐talus temperatures. In the mornings (05:15–08:15), temperatures below the talus were slightly warmer than above‐talus conditions, with higher minimum (mean°C below talus – mean°C above talus [standard deviation]: +2.50°C [1.69°C]), and maximum temperatures (+2.62°C [1.69°C]). During the warmest part of the day (13:00–16:00), the temperature‐damping effects of the below‐talus thermal refuge were most pronounced, exhibiting substantially lower minimum (−13.08°C [2.42°C]) and maximum temperatures (−15.44°C [1.94°C]). The evenings (19:00–21:00) produced higher minimum temperatures below‐talus (+2.75°C [1.95°C]), but lower maximums (−3.75°C [1.78°C]).

Sensitivity analyses of Niche Mapper's microclimate model revealed sufficient stability in response to input variability within ranges expected in the field, consistent with previous tests (Kearney et al. 2014a,b). Varying the specific heat of the talus by up to 30% (700–1000 J/kg K) produced a maximum 12% change in modeled surface temperatures and 10% change in temperatures 15 cm below the surface. Increasing wind speed from 0.1 to 1.5 m/sec decreased modeled temperatures at animal height (9 cm) by a maximum of 15%; further increases from 1.5 to 9 m/sec produced at most a 10% temperature decrease. Modifying cloud cover from 0 to 100% and aspect from south to north produced a maximum 9% and 2.5% decrease in temperature at animal height, respectively. The remaining inputs produced <2% changes in modeled temperature when varied within expected ranges.

The microclimate model also produced relatively robust estimates of above‐ and below‐talus temperatures when compared to field measurements (see Figs. S2–S5). Surface temperature estimates most closely matched observed when a −8°C/km altitudinal lapse rate was used to estimate air temperature and when wind speed was allowed to vary diurnally from 0 to 4 m/sec. Below‐talus temperature estimates fit field measurements closest using a modeled depth of 20 cm for the 1056 m site and 15 cm for the 2521 m site. The microclimate model assumed a solid below‐surface substrate (in contrast to porous talus deposits); thus, simulated depths were 100–200% less than the size of the rocks under which the loggers were placed.

### Metabolic chamber simulations

#### Sensitivity analyses for key animal model inputs

The effect of ambient temperature on metabolic rate was most heavily influenced by the core temperature, body posture, size, and fur properties of the modeled pika. Increasing core temperature from 38 to 43°C produced 8–25% higher metabolic demands (W/g), depending on ambient temperature. Changing posture from a 3:2 to 3:1 ellipsoid produced ~10% higher metabolic demands in a 150 g pika at temperatures ranging from −15 to 30°C. At cooler temperatures, pikas with lower mass (e.g., 109 g), and thus smaller volume, were at an energetic disadvantage because they were required to generate proportionally more metabolic heat to maintain core temperatures. However, above 22°C, smaller pikas had an energetic advantage in that they were able to avoid overheating at higher temperatures. Additionally, summer fur allowed pikas of all sizes to stay in their thermal neutral zone at temperatures 4°C higher than those with winter fur (see Fig. S6). Increasing fur density from 250 to 3000 hairs/cm^2^ produced 61% lower metabolic rates at 0°C; further increases from 3000 to 15,000 hairs/cm^2^ produced 15% lower rates. Increasing O_2_ extraction efficiency from 5 to 30% produced up to 6% lower metabolic rates, while increasing flesh thermal conductivity from 0.5 to 2.8 (W/mK) increased metabolic rates by up to 13%. Finally, when we modeled a constant solar input of 500 W/m^2^ directly overhead, increasing pelt reflectivity from 15 to 90% produced 15% higher metabolic rates.

#### Comparison to metabolic chamber measurements

**Figure 2:**
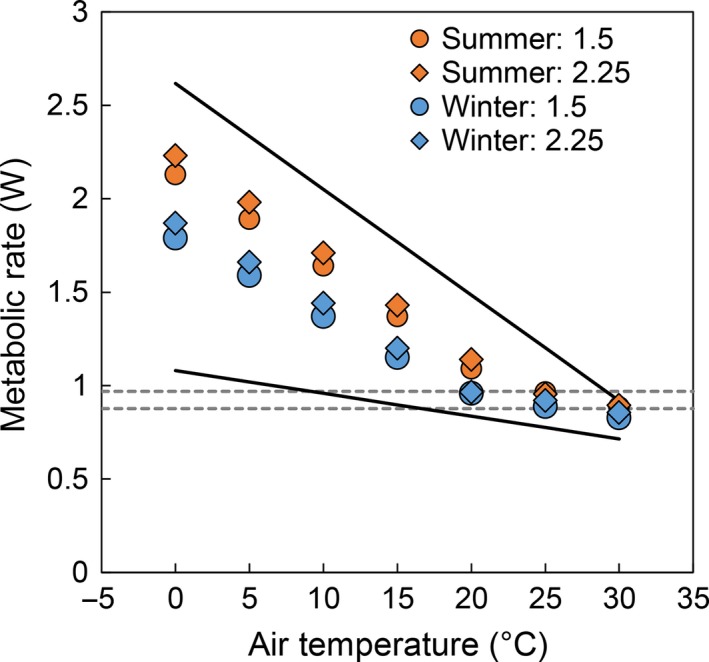
Niche Mapper simulations of American pika metabolic rate (W) in a metabolic chamber at a range of temperatures, animal shape (ellipsoid a:b ratio 1.5 and 2.25) and fur properties. Solid lines represent the range of empirical minimum metabolic rates for pikas measured by MacArthur and Wang (1973). The dashed lines represent the target BMR range of 0.923 W ± 5%.

Metabolic rate simulations for 109 g pikas fell within the range empirically derived by MacArthur and Wang (1973) for a variety of body postures and fur properties (Fig. 2). Niche Mapper simulated an upper critical temperature threshold (FMR = BMR) of ~25°C, which is similar to observed values (MacArthur and Wang 1973; Smith 1974).

### Field observations of pika activity in GNP

**Figure 3:**
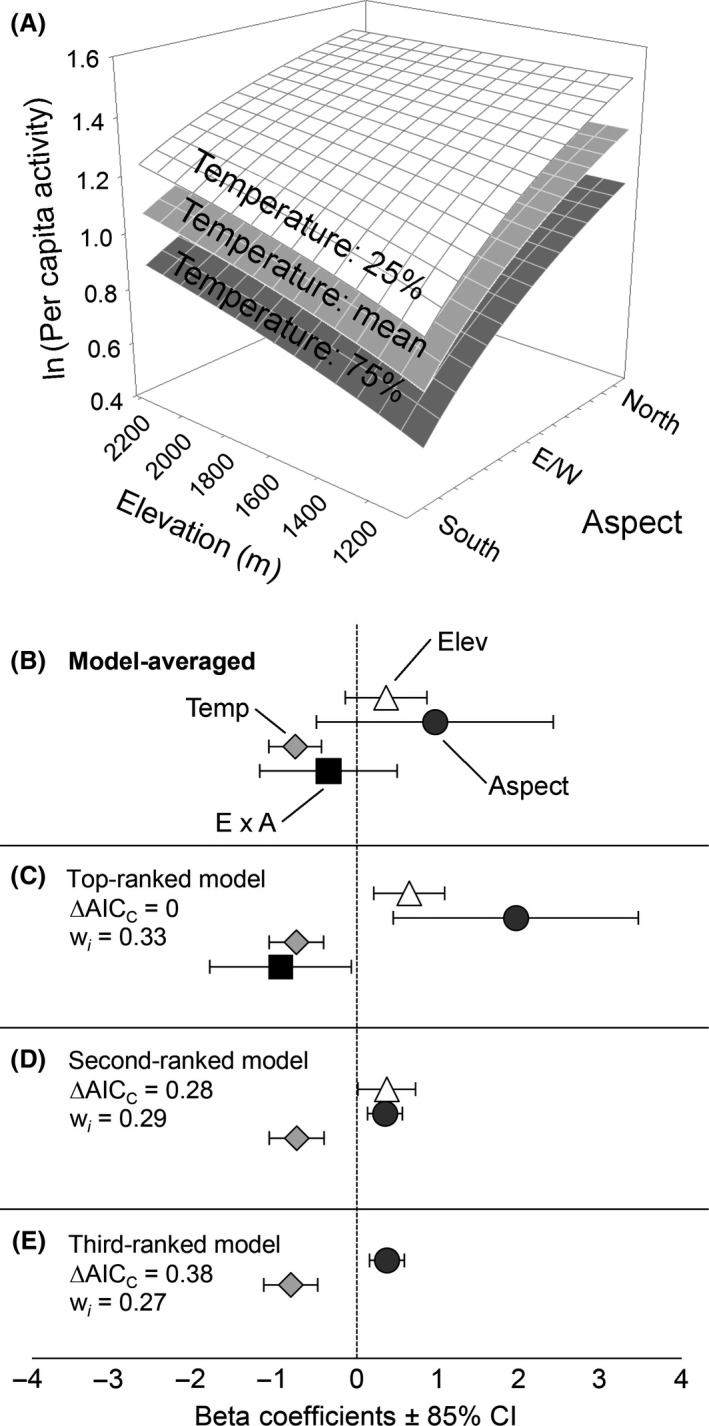
Relationship between field‐based observations of pika activity, minimum above‐talus temperature, elevation, and slope aspect. The top graph (A) shows model‐averaged predicted regression surfaces at three temperatures: 25% quartile, mean, and 75% quartile. Beta coefficients and 85% confidence intervals (Arnold 2010) are shown (B) following model averaging, and for the (C) top‐ranked model, (D) second‐ranked model, and (E) third‐ranked model.

There were three closely competing models within 0.4 AIC that combined for ~90% of the total AIC weight; the top model, {ln(*e*) + ln(*a*) + ln(*t*) + [ln(*e*) × ln(*a*)]}, contained all covariates that were considered and therefore had the most parameters (*k* = 5), followed by more parsimonious models [ln(*e*) + ln(*a*) + ln(*t*)] and [ln(*a*) + ln(*t*)] (see Table S8). We calculated model‐averaged regression coefficients and unconditional 85% confidence intervals for parameters in these models to reduce model selection bias (Burnham and Anderson 2002; Arnold 2010) and produce conservative estimates of effect sizes (Fig. 3A and B) because some estimates varied to an appreciable extent among models (Fig. 3C and E). Model‐averaged parameter estimates suggested that pikas were generally more active at higher elevations (${\hat{\overset{¯}{\beta }}}_{\text{ln}(e)}$ = 0.36, SE = 0.35), on north‐facing slopes (${\hat{\overset{¯}{\beta }}}_{\text{ln}(a)}$ = 0.96, SE = 1.00), and when minimum above‐talus temperatures were cooler (${\hat{\overset{¯}{\beta }}}_{\text{ln}(t)}$ = −0.76, SE = 0.22) (Fig. 3A). Elevation and aspect were linked by a negative interaction (${\hat{\overset{¯}{\beta }}}_{\left[\text{ln}(e)\times \text{ln}(a)\right]}$ = −0.35, SE = 0.59), which suggested that pikas on south‐facing slopes were less active at low elevations than at high elevations, but pikas were more active above the talus on north‐facing slopes regardless of elevation (Fig. 3A).

**Figure 4:**
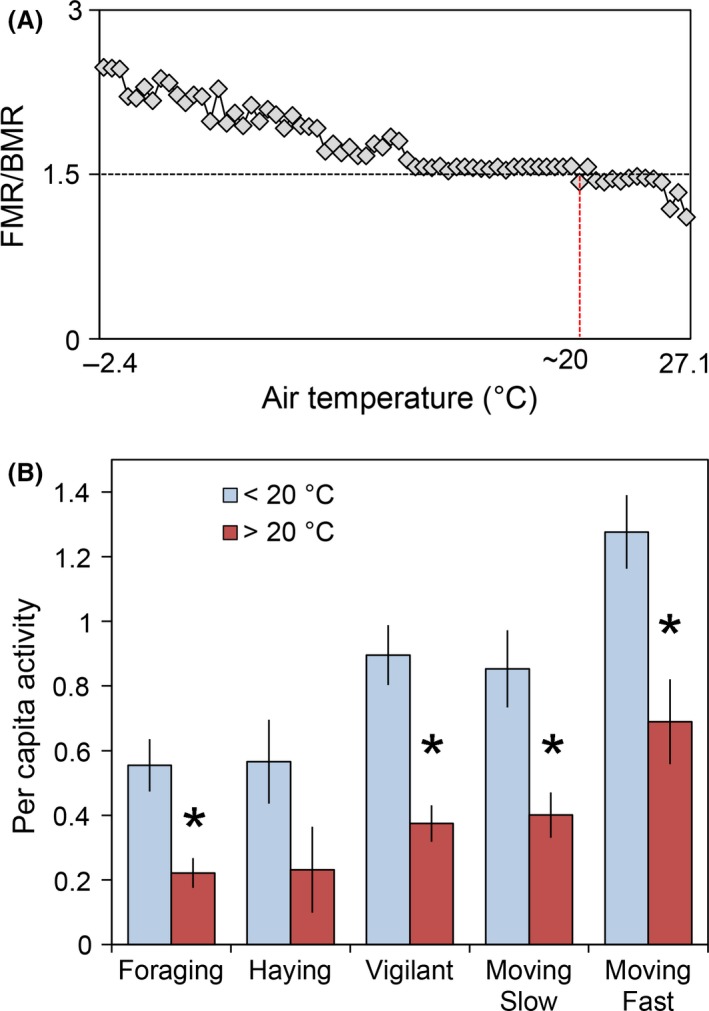
The top panel (A) shows FMR/BMR ratios across a range of temperatures. The red vertical line denotes the temperature threshold at which modeled 150 g pikas could not maintain an FMR/BMR above 1.5. The bottom panel (B) shows the per capita frequency of pika behaviors when minimum above‐talus temperature was above or below 20°C during field observations in GNP. Error bars represent ±1 SE. *Denotes a significant difference between the two groups.

In addition to our model selection‐based approach evaluating aggregate pika activity in relation to minimum above‐talus temperature and site‐level variables, we used an ANOVA‐based approach to evaluate whether minimum above‐talus temperature and time of day were significantly related to specific pika behaviors. We found that pikas were significantly more active in all behavior categories (except for haying) when minimum above‐talus temperature was below 20°C (all *P* < 0.01), which corresponded with a metabolic threshold predicted by Niche Mapper (Fig. 4). Calls per occupant were influenced by time of day and temperature; there were 14.7 fewer calls per occupant during midday than during morning or evening (*P* < 0.01) and 0.5 fewer calls per occupant for every 1°C (*P* < 0.01, *R* ^2 ^= 0.08). Data were also collected on whether or not pikas were exposed to direct sunlight while making short or long call vocalizations. Long calls are typically made by males and probably related to mating (Smith and Weston 1990). Only one of 149 (0.67%) recorded long calls and 400 of 6699 (6%) of short calls were made while pikas were exposed to direct sunlight.

### Field simulations

#### Comparing modeled and observed behavior

**Figure 5:**
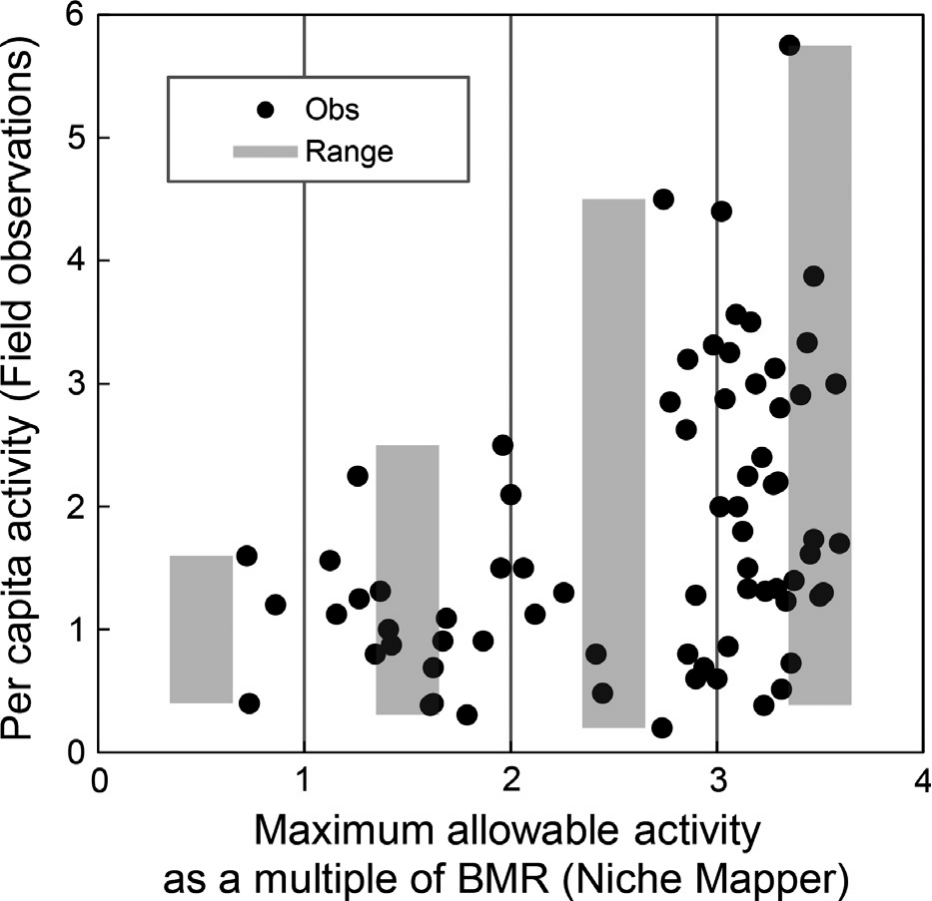
Comparison of Niche Mapper predicted maximum allowable activity at field sites (*x*‐axis) with observed pika activity frequency at those same sites in the field (*y*‐axis).

Niche Mapper's activity level predictions were similar to observations at field sites in GNP. Predicted activity levels, quantified by the ratio of maximum allowable FMR to BMR, were lowest when temperatures were high and increased as temperatures dropped. Above 20°C, modeled 150 g pikas began physiologically thermoregulating (vasodilation, raising body temperature) in order to maintain a target metabolic rate of 1.5 ×  their BMR, which corresponds to the minimum value necessary for activity (Fig. 4A). Additionally, increasing predicted allowable activity levels corresponded to the highest rates and variance of observed per capita pika activity (Fig. 5).

Using 1.5 FMR/BMR as the minimum metabolic ratio for activity, a simulated 120 g pika in GNP was able to remain active above the talus during all available diurnal hours of the day (June and July = 21, August = 19 h), regardless of elevation or pelage (summer vs. winter). However, a 150 g pika with winter pelage faced physiological limitations that reduced allowable activity time by 19.0% in July at 1500 m, 4.8% in July at 1750 m, and 15.8% in August at 1500 m.

#### Simulating the below‐talus thermal refuge

**Figure 6:**
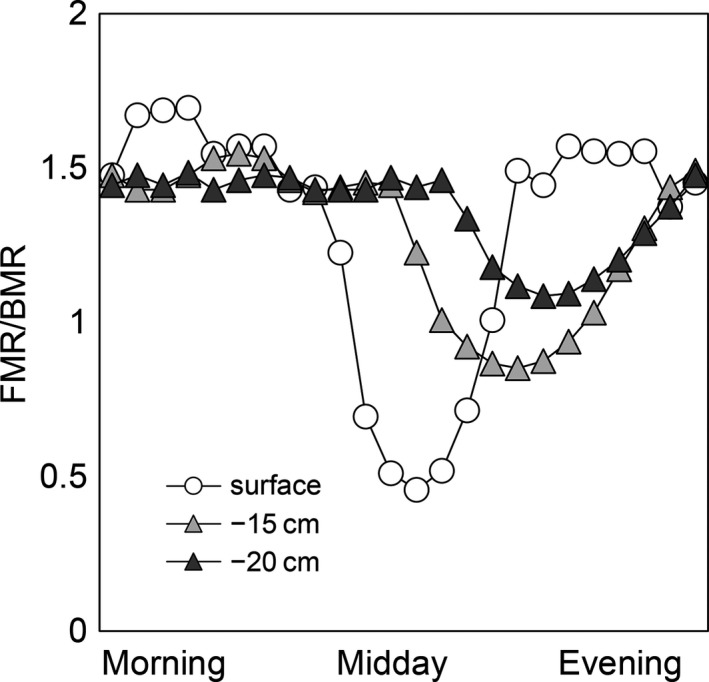
Niche Mapper simulations of FMR/BMR for a pika at three depths (surface, 15 cm below‐surface, and 20 cm below‐surface) during a heat wave. Activity cannot occur below 1.5 FMR/BMR.

We simulated a heat wave with temperatures ~10°C higher than current July means at low elevations in GNP to explore how the talus might buffer a pika from acute heat stress. For several consecutive hours in the middle of the day, the model pika was unable to maintain even its BMR without overheating, suggesting death from hyperthermia. However, at sufficient depth below the surface, the model pika could retreat and maintain its BMR for all hours of the day without overheating (Fig. 6).

#### Simulations under a future warming scenario

**Figure 7:**
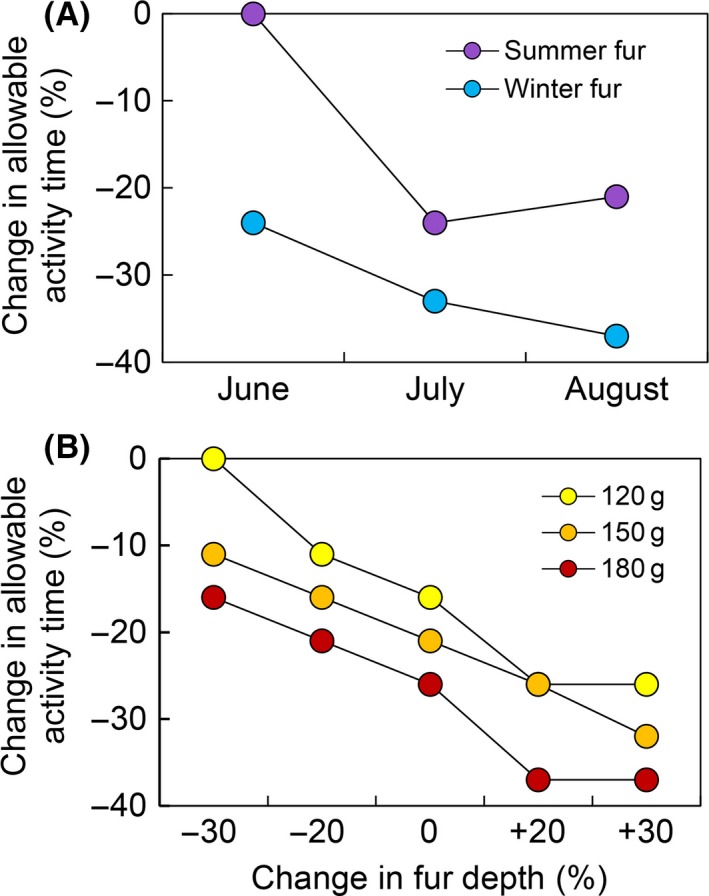
Niche Mapper simulations of percent (%) change in metabolically allowable daily above‐talus activity time by year 2100 for a pika at 1500 m elevation in Glacier National Park, MT, for (A) a 150 g pika during summer months in summer fur and winter fur and (B) for different sizes of pikas with varying fur depths, during the month of August.

A 2100 warming scenario of +5°C resulted in restrictions in allowable activity time (diurnal + crepuscular hours) for simulated pikas in GNP, especially when pikas had a higher body mass and thicker fur. Simulations suggested that allowable activity time for a 120 g pika with summer fur in year 2100 will decline by 9.5%, but only in July and at a low elevation (1500 m). However, allowable activity time for a 150 g pika with summer fur declined by 10.5–23.8% in July and August at both 1500 m (Fig 7A) and 1750 m elevation sites.

With winter fur, potential activity time for a 150 g pika declined under the future warming scenario at elevations below 2500 m. In June, when many pikas may still have winter fur, allowable activity time declined by 23.8% and 4.8% at 1500 m and 1750 m, respectively. Further investigation of the impacts of interactions between body size and fur properties on allowable activity for a pika at low elevation (1500 m) in GNP in year 2100 revealed no activity restrictions for a 120 g pika with 30% shorter and shallower fur (compared to the measured specimen), but 37% fewer activity hours for a 180 g pika with 20% longer and thicker fur (Fig. 7B).

## Discussion

Our understanding of animal species' resilience to warming temperatures is a critical component of our ability to predict future range shifts and extirpations, and conserve biodiversity. Animals employ an array of behavioral strategies to avoid hyperthermia, such as changing body posture, seeking cooler microhabitats, reducing locomotion, and decreasing energy intake (Terrien et al. 2011). Mammals in particular have the ability to regulate body temperature through fur properties, for example, by changing their seasonal molt timing. We examined some of these factors by presenting a coupled biophysical endotherm and microclimate model and comparing the results to empirical temperature and behavior data for a species, the American pika, whose ability to persist under future climate warming is unclear.

Although recent work has shown that pika extirpations during the past 35–55 years in the Great Basin were correlated with high mean summer temperatures and both extreme heat in the summer and extreme cold in the winter (Beever et al. 2003, 2010), the mechanisms driving these losses are unclear. A critical gap exists regarding our understanding of how climate and pika physiology interact to affect behavior. Thus, it is unclear how pika physiological tolerances and behavioral flexibility will impact their individual and population‐level responses to rapid climate change. To date, the most thorough studies of pika behavior relative to temperature found that pikas were less active during midday, especially at lower elevation sites (Smith 1974), and that activity durations were short. It was presumed that pikas used short activity bouts to limit heat accumulation and used the sub‐talus microclimate to dissipate heat (MacArthur and Wang 1974). Others have reported midday activity decreases in GNP (Barash 1973), as well as the Sierra Nevada (Severaid 1955) and Great Basin (Hall 1946). However, research in Colorado (Broadbooks 1965; Krear 1965), and the high Sierra (Grinnell and Storer 1917) suggested that pikas remained active throughout the day. Thus, analysis of the mechanisms driving pika extirpations requires a greater biophysical understanding of pikas as well as their capacity to behaviorally thermoregulate.

### Microclimate data

Uncertainty in a temperature‐occupancy threshold for pikas in the literature (e.g., Beever et al. 2010; Millar and Westfall 2010; Smith and Nagy 2015) may stem from a reliance on broad‐scale weather data in place of pika‐relevant microclimate data (Potter et al. 2013). Mean free atmospheric lapse rates are too coarse to accurately characterize temperature over complex terrain and at fine spatial scales (Minder et al. 2010). Instead, regional monthly and daily surface temperature lapse rates such as those we calculated are more appropriate (Blandford et al. 2008), especially when combined with a microclimate model such as Niche Mapper's. We identified relatively large variation in the relationship between the microclimates available to pikas and elevation in GNP; when consistent, this lapse rate had a 1–3°C greater magnitude than expected for the region (Ray et al. 2010). Pikas could potentially exploit this variability during warmer summers by increasing their activity during short periods of favorable conditions or even increasing nocturnal activity (Severaid 1955; Smith 1974). We recommend caution when applying large‐scale climate data and model predictions, such as lapse rate estimates, in mountainous terrain.

The thermal refuge beneath the talus plays a critical role in pika thermoregulation. We found that it provided a buffer of up to 20°C during the hottest times of day, which is consistent with the literature (MacArthur and Wang 1974; Varner and Dearing 2014), although it can be more pronounced in the presence of subsurface ice features (Wilkening et al. 2015). This buffer allows pikas to remain active under the talus surface and avoid acute heat stress or death on hot days (Fig. 6). However, our simulations showed that relatively shallow talus may provide insufficient thermal refuge during heat waves, especially in the late afternoon and early evening, such that microclimates become unsuitable both above and below the talus.

### Metabolic chamber simulations

We demonstrated that Niche Mapper accurately predicted pika metabolic rates from metabolic chamber experiments across a wide range of temperatures (Fig. 2). Sensitivity analyses revealed that fur properties, animal size, and ambient wind speed had relatively large physiological, and thus behavioral, consequences. These factors are rarely considered in studies of pika behavior and occurrence, but our work suggests that they may play a critical role in pika persistence in a warming climate.

### Behavior observations

We found pika activity was lowest when temperatures were highest, which is consistent with the literature (e.g., Barash 1973; MacArthur and Wang 1974; Smith 1974). Niche Mapper identified a limiting temperature threshold of 20°C for a 150 g pika with summer fur, above which we observed restrictions in most above‐talus behaviors (Fig. 4). We found no correlation between behavior and temperature below this threshold, suggesting that other factors may exert stronger influences on behavior below 20°C. For example, the predominance of short activity bouts may play a role in predator avoidance. Pika calls, which were negatively correlated with temperature, probably serve as a conspecific territorial defense mechanism and a predator alarm (Conner 1983), but may also increase individual predation risk by revealing the caller's location (Smith and Ivins 1983). Our results suggest that temperature may modulate an individual's cost–benefit curve for making vocalizations as well as restrict above‐talus activity.

Behavioral observations revealed that, in addition to temperature, site elevation and aspect combined to impact pika activity in complex ways. Pikas at higher elevations and north‐facing slopes were generally most active (Fig. 3). While high elevations and north‐facing slopes may contribute to the cooler conditions preferred by pikas, they also may contribute to differences in seasonality and phenology that are more pronounced at high latitudes. Later snowmelt and earlier, more frequent snowfall at high‐elevation north‐facing sites may constrain or shift the timing of the growing season, which could limit food availability. Pikas inhabiting such sites may be compelled to increase foraging and/or haying bouts per unit time relative to other sites to meet caloric demands for metabolic maintenance, reproduction, and food caching.

The seasonality of haying and breeding activities in pikas may have considerable impacts on observations of their behavior. Compared to higher elevations, pikas at lower elevations start and cease haying sooner, and breed up to 6 weeks sooner (Smith 1974), which could be driven by elevational gradients in vegetation senescence (Smith 1974; Huntly et al. 1986). Additionally, pikas change their preferred forage throughout the season in response to changes in plant toxins and protein content, which may compel them to accelerate haying at different times (Dearing 1997). We conducted our field observations during a 10‐day period to reduce behavioral changes due to seasonality. However, spatial differences in seasonality across sites may have contributed to the support for elevation, aspect, and elevation × aspect terms as predictors of pika activity (Fig. 3).

### Field simulations and activity observations

We used simulations to demonstrate pika physiological activity constraints over a range of temperatures, elevations, and fur properties. The metabolic consequences of thermoregulation are reflected in the FMR/BMR ratio, which constrains animals at low and high temperature extremes. Pikas must maintain an FMR of at least 1.5 ×  BMR to remain active above the talus, but as low as possible above this threshold to conserve energy. While our summer‐month simulations produced FMR/BMR ratios no greater than 3.0, higher ratios could occur in winter when pikas face hypothermia.

We demonstrated that fur properties, molt timing, and animal size have significant metabolic impacts on pikas, thereby constraining activity time (Fig. 7). The role of fur properties in pika behavior, morphology, and physiology has, to date, been unexamined. To our knowledge, only three studies have documented pika fur properties. Krear (1965) and Howell (1924) documented two annual molts, noting that summer pelage is generally worn for < 2 months and that winter fur is up to twice the length of summer fur, while Severaid (1955) examined the effect of reproductive status on molt timing. However, the extent to which subpopulations inhabiting disparate climatic regions (or microclimates on the same mountain) vary in fur properties and molt timing is not well known. Our simulations suggested that plasticity in molt timing, to a certain degree, may reduce activity restrictions predicted under future warming scenarios. In particular, pikas that shed their winter pelage sooner could be less restricted in early growing season activity.

Geographic variation in size and pelage can have substantial biophysical impacts on endotherms (Briscoe et al. 2014). Range‐wide variation in pika body size is poorly documented, yet we demonstrated that body size could affect potential activity time under climate change. Smith and Weston (1990) reported interpopulation variation in pika mean body mass ranging from 121 to 176 g, but their sample was relatively limited in size and geographic extent. In addition to limiting activity (Fig. 7), animal size may interact with temperature to impact dispersal. Smith (1974) suggested that pikas at low elevations were limited primarily by low colonization success associated with higher temperatures, and that furthermore these pikas were forced to disperse during the hottest times of the season. However, smaller dispersing juveniles (Millar and Tapper 1973) can dissipate heat more efficiently and remain active at higher temperatures (Fig. 7). More range‐wide data on variation in fur properties, molt timing, and animal size, especially for at‐risk subpopulations, is critical to improve our understanding of pika thermoregulation and potential responses to future warming.

The nocturnal activity of pikas warrants further investigation, especially at warmer low‐elevation sites (e.g., Beever et al. 2008). Little is known about nocturnal activity in pikas; nocturnal activity may be restricted to certain regions and subpopulations, or may be a behavioral adaptation available to all pikas. While several occurrences of nocturnal activity and vocalizations have been documented in the Sierra Nevada and GNP (Smith 1974; LMH *pers. obs*.), Krear (1965) observed no nocturnal activity during extensive observations in Colorado. The risk of depredation by efficient nocturnal predators such as owls and weasels could play a role in restricting pika nocturnal activity.

The results presented here combine mechanistic biophysical and empirical correlative approaches to investigate the temperature‐limitations of American pikas. The approaches complemented one another, and together formed a more complete understanding of pika heat tolerance and thermoregulatory options. The empirical observations and correlative analyses provided critical support for the mechanistic model's predictions while also identifying important factors, in this case elevation, aspect, and possibly seasonality that were not modeled biophysically. In turn, the biophysical model provided mechanistic support for our understanding of the drivers of pika behaviors observed by us and other researchers. Future research could evaluate allowable activity time across the pika's range to model spatial patterns of occupancy and extirpation.

Niche Mapper simulations allowed us to identify additional significant factors and generate hypotheses for investigation by future empirical studies. In the case of American pikas, the complex relationship between fur properties, body size, behavior, and microclimate structure may be critical to species persistence. For instance, we found that smaller pikas with shorter fur were better able to tolerate higher temperatures and remain active longer than larger pikas with thicker fur. Rising temperatures will present selective pressures on pika populations relative to the characteristics that we identified. Approaches that combine correlative and mechanistic analyses are becoming increasingly necessary in an age of rapid climate change.

## Conflict of Interest

None declared.

## Data Accessibility

Data are available from L. Moyer‐Horner upon request.

## Supporting information


**Figure S1.** Graphical representations of the supported log‐linear relationships between environmental variables and field‐based observations of pika activity in Glacier National Park, MT during the summers of 2008 and 2009.
**Figure S2**. Comparison of NicheMapper microclimate model surface temperature estimates to measured surface temperatures in GNP at 1056 m elevation, between July 15–July 24, 2009.
**Figure S3**. Comparison of NicheMapper microclimate model surface temperature estimates to measured surface temperatures in GNP at 2521 m elevation, between July 15–July 24, 2009.
**Figure S4**. Comparison of NicheMapper microclimate model below‐talus temperature estimates to measured below‐talus temperatures in GNP at 1056 m elevation, between July 15–July 24, 2009.
**Figure S5**. Comparison of NicheMapper microclimate model below‐talus temperature estimates to measured below‐talus temperatures in GNP at 2521 m elevation, between July 15–July 24, 2009.
**Figure S6**. Niche Mapper simulations of an American pika (*Ochotona princeps*) in a metabolic chamber, across a range of temperatures, varying the size and fur properties of the animal.
**Table S1.** Properties used to parameterize the biophysical model for an American pika (*Ochotona princeps*).
**Table S2.** Model selection results for the relationship between relevant temperature covariates and field‐based observations of pika activity in Glacier National Park, MT during the summers of 2008 and 2009.
**Table S3.** Set of 10 a priori hypotheses relating a suite of environmental variables (*e*, elevation; *a*, aspect; *t*, minimum above talus temperature) to field‐based observations of pika activity in Glacier National Park, MT during the summers of 2008 and 2009.
**Table S4.** Standard microclimate model inputs for Niche Mapper simulations of American pikas (*Ochotona princeps*) in Glacier National Park, Montana, USA.
**Table S5.** Temperature (°C) data from time 0700 on the dates indicated in 2008, 1 m below the talus surface at seven sites in Glacier National Park, Montana.
**Table S6.** Temperature (°C) data from time 1400 on the dates indicated in 2008 and 1 m below the talus surface at seven sites in Glacier National Park, Montana.
**Table S7.** Temperature (°C) data from time 2000 on the dates indicated in 2008 and 1 m below the talus surface at seven sites in Glacier National Park, Montana.
**Table S8.** Model selection results for analysis of field‐based observations of pika behavior (per capita pika activity) in Glacier National Park, Montana during the summers of 2008 and 2009.
