# Supplementary material for: Modeling behavioral thermoregulation in a climate change sentinel
Source: Ecol Evol. 2015 Nov 24;5(24):5810–22. doi: 10.1002/ece3.1848 (PMC4717337; doi:10.1002/ece3.1848)
Supplement: Supplementary file 1 — Figure S1. Graphical representations of the supported log‐linear relationships between environmental variables and field‐based observations of pika activity in Glacier National Park, MT during the summers of 2008 and 2009. Figure S2. Comparison of NicheMapper microclimate model surface temperature estimates to measured surface temperatures in GNP at 1056 m elevation, between July 15–July 24, 2009. Figure S3. Comparison of NicheMapper microclimate model surface temperature estimates to measured surface temperatures in GNP at 2521 m elevation, between July 15–July 24, 2009. Figure S4. Comparison of NicheMapper microclimate model below‐talus temperature estimates to measured below‐talus temperatures in GNP at 1056 m elevation, between July 15–July 24, 2009. Figure S5. Comparison of NicheMapper microclimate model below‐talus temperature estimates to measured below‐talus temperatures in GNP at 2521 m elevation, between July 15–July 24, 2009. Figure S6. Niche Mapper simulations of an American pika (Ochotona princeps) in a metabolic chamber, across a range of temperatures, varying the size and fur properties of the animal. Table S1. Properties used to parameterize the biophysical model for an American pika (Ochotona princeps). Table S2. Model selection results for the relationship between relevant temperature covariates and field‐based observations of pika activity in Glacier National Park, MT during the summers of 2008 and 2009. Table S3. Set of 10 a priori hypotheses relating a suite of environmental variables (e, elevation; a, aspect; t, minimum above talus temperature) to field‐based observations of pika activity in Glacier National Park, MT during the summers of 2008 and 2009. Table S4. Standard microclimate model inputs for Niche Mapper simulations of American pikas (Ochotona princeps) in Glacier National Park, Montana, USA. Table S5. Temperature (°C) data from time 0700 on the dates indicated in 2008, 1 m below the talus surface at seven sites in Glacier National Pa [file ECE3-5-5810-s001.docx]

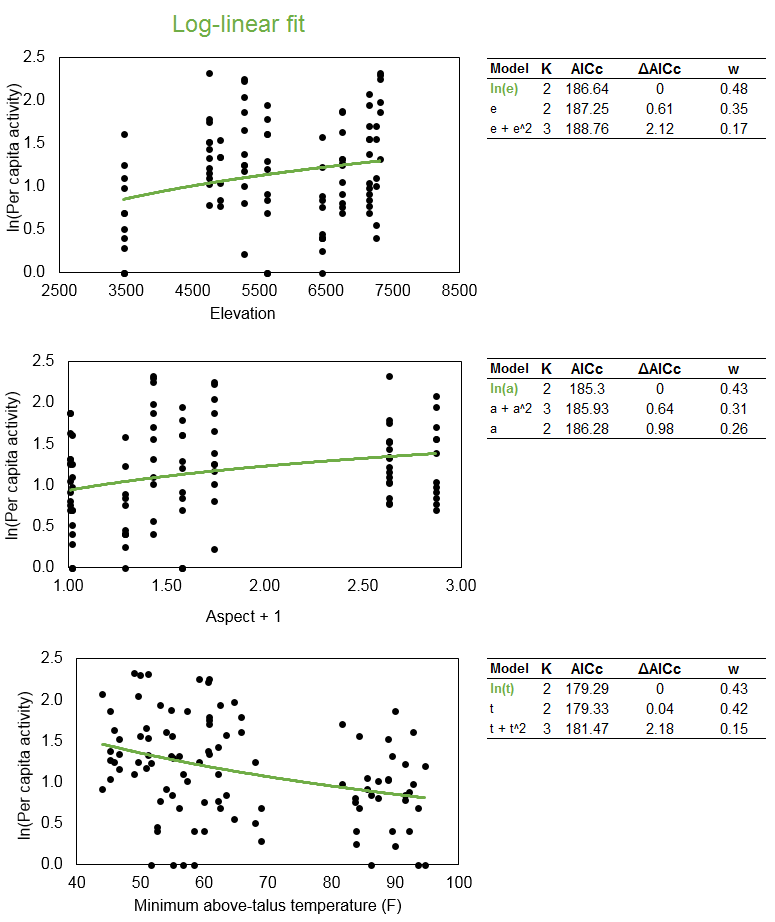


**Figure S1.** Graphical representations of the supported log-linear relationships between environmental variables and field-based observations of pika activity in Glacier National Park, MT during the summers of 2008 and 2009.

**Figure S2**. Comparison of NicheMapper microclimate model surface temperature estimates to measured surface temperatures in GNP at 1056 m elevation, between July 15 – July 24, 2009. NicheMapper estimates were derived from temperatures measured at Kalispell International Airport, 961 m elevation and ~30km from GNP. Wind speed was allowed to fluctuate diurnally from 0 – 4 m/s or held constant at 2 m/s. Altitudinal lapse rates (ALR) of -6,- 8, and -10 ºC/km were applied for comparison. The temperature logger was shaded by a mountain during the mornings and near a creek, likely causing of the delayed rise in temperature compared to model predictions.

**Figure S3**. Comparison of NicheMapper microclimate model surface temperature estimates to measured surface temperatures in GNP at 2521 m elevation, between July 15 – July 24, 2009. NicheMapper estimates were derived from temperatures measured at Kalispell International Airport, 961 m elevation and ~30km from GNP. Wind speed was allowed to fluctuate diuranally from 0 – 4 m/s or held constant at 2 m/s. Altitudinal lapse rates (ALR) of -6,- 8, and -10 ºC/km were applied for comparison.

**Figure S4**. Comparison of NicheMapper microclimate model below-talus temperature estimates to measured below-talus temperatures in GNP at 1056 m elevation, between July 15 – July 24, 2009. NicheMapper estimates were derived from temperatures measured at Kalispell International Airport, 961 m elevation and ~30km from GNP. Temperatures were modeled at depths ranging from 5 – 30 cm below the talus surface with an altidunal lapse rate of -8 ºC/km.

**Figure S5**. Comparison of NicheMapper microclimate model below-talus temperature estimates to measured below-talus temperatures in GNP at 2521 m elevation, between July 15 – July 24, 2009. NicheMapper estimates were derived from temperatures measured at Kalispell International Airport, 961 m elevation and ~30km from GNP. Temperatures were modeled at depths ranging from 5 – 30 cm below the talus surface with an altidunal lapse rate of -8 ºC/km.

**Figure S6**. Niche Mapper simulations of an American pika (*Ochotona princeps*) in a metabolic chamber, across a range of temperatures, varying the size and fur properties of the animal. Winter fur was 50% longer and deeper than summer fur.

**Table S2.** Model selection results for the relationship between relevant temperature covariates and field-based observations of pika activity in Glacier National Park, MT during the summers of 2008 and 2009.

| Model | AICc | ∆AICc | w_i_ | *k* |
| --- | --- | --- | --- | --- |
| T_MIN_ above | 179.06 | 0 | 0.65 | 2 |
| T_AVG_ above | 181.27 | 2.22 | 0.22 | 2 |
| T_AVG_ below | 184.20 | 5.14 | 0.05 | 2 |
| T_MAX_ above | 184.57 | 5.52 | 0.04 | 2 |
| T_MAX_ below | 186.20 | 7.14 | 0.02 | 2 |
| T_MIN_ below | 186.28 | 7.22 | 0.02 | 2 |
| null | 189.70 | 10.65 | 0 | 1 |

**Table S3.** Set of 10 *a priori* hypotheses relating a suite of environmental variables (e – elevation, a – aspect, t – minimum above talus temperature) to field-based observations of pika activity in Glacier National Park, MT during the summers of 2008 and 2009.

| Model | Structure | Prediction |
| --- | --- | --- |
| e | β_0_ + β_e_ + ϵ | β_e_ > 0 |
| a | β_0_ + β_a_ + ϵ | β_a_ > 0 |
| t | β_0_ + β_t_ + ϵ | β_t_ < 0 |
| e + a | β_0_ + β_e_ + β_a_ + ϵ | β_e_ > 0, β_a_ > 0 |
| e + t | β_0_ + β_e_ + β_t_ + ϵ | β_e_ > 0, β_t_ < 0 |
| a + t | β_0_ + β_a_ + β_t_ + ϵ | β_a_ > 0, β_t_ < 0 |
| e + a + t | β_0_ + β_e_ + β_a_ + β_t_ + ϵ | β_e_ > 0, β_a_ > 0, β_t_ < 0 |
| e + a + e × a | β_0_ + β_e_ + β_a_ + β_e × a_ + ϵ | β_e_ > 0, β_a_ > 0, β_e × a_ < 0 |
| e + a + t + e × a | β_0_ + β_e_ + β_a_ + β_t_ + β_e × a_ + ϵ | β_e_ > 0, β_a_ > 0, β_t_ < 0, β_e × a_ < 0 |
| intercept-only | β_0_ + ϵ | - |

**Table S5.** Temperature (ºC) data from time 0700 on the dates indicated in 2008, 1 m below the talus surface at seven sites in Glacier National Park, Montana. Sites are denoted by their elevations (m). Each date was fitted with a linear regression. The slope of that line indicates the lapse rate (ºC/km). R-squared values are provided to show the fit of the line to the data. The mean slope for all eight dates is given.

| **Site Elevation** | **Jul-28** | **Aug-4** | **Aug-11** | **Aug-18** | **Aug-25** | **Sep-1** | **Sep-8** | **Sep-15** |  |
| --- | --- | --- | --- | --- | --- | --- | --- | --- | --- |
| 2274 | 9.7 | 6.8 | 4.2 | 18.6 | 12.5 | -1.3 | 1.4 | 9.3 |  |
| 2272 | 9.2 | 6.7 | 4.9 | 17.4 | 12.3 | -0.2 | 1.9 | 10.3 |  |
| 2207 | 8.9 | 6.3 | 6.3 | 9.6 | 10.0 | 0.4 | 1.7 | 1.5 |  |
| 2054 | 11.1 | 8.7 | 6.5 | 15.7 | 12.3 | 1.3 | 3.9 | 7.1 |  |
| 2031 | 12.0 | 8.8 | 8.2 | 17.6 | 13.7 | 0.2 | 3.3 | 8.3 |  |
| 1765 | 14.0 | 7.4 | 9.4 | 16.3 | 14.8 | 4.1 | 3.3 | 5.8 |  |
| 1512 | 15.7 | 6.3 | 11.6 | 16.8 | 15.0 | 7.7 | 3.7 | 5.0 | **Mean** |
| **Slope (ºC/km)** | -8.81 | 0.10 | -8.87 | -1.37 | -4.83 | -10.65 | -2.71 | 3.50 | -4.21 |
| **R^2^** | 0.957 | 0.001 | 0.938 | 0.017 | 0.631 | 0.940 | 0.555 | 0.113 |  |

**Table S6.** Temperature (ºC) data from time 1400 on the dates indicated in 2008 and 1 m below the talus surface at seven sites in Glacier National Park, Montana. Sites are denoted by their elevation (m). Each date was fitted with a linear regression. The slope of that line indicates the lapse rate (ºC/km). R-squared values are provided to show the fit of the line to the data. The mean slope for all eight dates is given.

| **Site Elevation** | **Jul-28** | **Aug-4** | **Aug-11** | **Aug-18** | **Aug-25** | **Sep-1** | **Sep-8** | **Sep-15** |  |
| --- | --- | --- | --- | --- | --- | --- | --- | --- | --- |
| 2274 | 18.2 | 17.6 | 7.7 | 28.6 | 20.4 | 0.2 | 11.3 | 17.3 |  |
| 2272 | 12.2 | 11.4 | 8.7 | 22.1 | 15.4 | -0.1 | 8.8 | 15.3 |  |
| 2207 | 9.9 | 8.1 | 7.9 | 10.9 | 12.1 | 0.9 | 3.1 | 3.2 |  |
| 2054 | 15.2 | 14.1 | 11.5 | 19.4 | 12.6 | 3.3 | 4.1 | 7.1 |  |
| 2031 | 12.6 | 12.4 | 8.9 | 20.1 | 14.3 | 1.1 | 4.5 | 10.0 |  |
| 1765 | 22.7 | 21.9 | 14.5 | 33.6 | 23.5 | 5.1 | 14.4 | 19.7 |  |
| 1512 | 18.1 | 15.4 | 13.3 | 24.3 | 18.4 | 7.8 | 7.7 | 10.5 | **Mean** |
| **Slope (C/km)** | -9.02 | -7.42 | -8.31 | -9.47 | -6.18 | -10.04 | -3.07 | -1.79 | -6.91 |
| **R^2^** | 0.338 | 0.222 | 0.754 | 0.140 | 0.171 | 0.942 | 0.044 | 0.007 |  |

**Table S7.** Temperature (ºC) data from time 2000 on the dates indicated in 2008 and 1 m below the talus surface at seven sites in Glacier National Park, Montana. Sites are denoted by their elevation (m). Each date was fitted with a linear regression. The slope of that line indicates the lapse rate (ºC/km). R-squared values are provided to show the fit of the line to the data. The mean slope for all eight dates is given.

| **Site Elevation** | **Jul-28** | **Aug-4** | **Aug-11** | **Aug-18** | **Aug-25** | **Sep-1** | **Sep-8** | **Sep-15** |  |
| --- | --- | --- | --- | --- | --- | --- | --- | --- | --- |
| 2274 | 18.5 | 18.0 | 9.3 | 27.3 | 16.2 | 0.2 | 8.8 | 14.6 |  |
| 2272 | 13.6 | 12.8 | 9.6 | 22.3 | 12.3 | 1.1 | 7.6 | 12.1 |  |
| 2207 | NA | 8.6 | 9.1 | 13.3 | 11.0 | 2.0 | 3.1 | 2.7 |  |
| 2054 | 19.8 | 19.9 | 12.6 | 25.2 | 15.4 | 3.9 | 7.6 | 10.5 |  |
| 2031 | 14.9 | 14.5 | 11.2 | 23.9 | 15.4 | 2.2 | 5.6 | 11.1 |  |
| 1765 | 18.9 | 17.7 | 14.2 | 25.9 | 18.9 | 5.8 | 10.3 | 12.9 |  |
| 1512 | 21.6 | 19.6 | 16.5 | 28.0 | 16.8 | 8.4 | 9.5 | 11.8 | **Mean** |
| **Slope (ºC/km)** | -9.14 | -7.84 | -9.58 | -8.42 | -6.21 | -9.96 | -4.56 | -2.70 | -7.30 |
| **R^2^** | 0.441 | 0.292 | 0.948 | 0.231 | 0.435 | 0.946 | 0.274 | 0.041 |  |

**Table S8.** Model selection results for analysis of field-based observations of pika behavior (per capita pika activity) in Glacier National Park, Montana during the summers of 2008 and 2009

| Model | AIC_C_ | ∆AIC_C_ | *w*_i_ | *k* |
| --- | --- | --- | --- | --- |
| ln(e) + ln(a) + ln(t) + [ln(e) × ln(a)] | 174.8 | 0 | 0.33 | 5 |
| ln(e) + ln(a) + ln(t) | 175.1 | 0.28 | 0.29 | 4 |
| ln(a) + ln(t) | 175.2 | 0.38 | 0.27 | 3 |
| ln(e) + ln(t) | 178.4 | 3.63 | 0.05 | 3 |
| ln(t) | 179.3 | 4.49 | 0.04 | 2 |
| ln(e) + ln(a) + [ln(e) × ln(a)] | 182.9 | 8.14 | 0.01 | 4 |
| ln(e) + ln(a) | 182.9 | 8.14 | 0.01 | 3 |
| ln(a) | 185.3 | 10.49 | 0 | 2 |
| ln(e) | 186.6 | 11.83 | 0 | 2 |
| intercept-only | 190.1 | 15.33 | 0 | 1 |

**Table S1.** Properties used to parameterize the biophysical model for an American pika (*Ochotona princeps*)

| **Parameter (units)** | **Value** | **Source** |
| --- | --- | --- |
| Weight (g) | 150 | [1]: Mean body mass for this species 121-176 grams. |
| Basal metabolic rate (W) | 1.28 | [2]: Measured BMR on 16 pikas averaging 109 grams was 0.00855 W/g |
| Core temperature (ºC)  Min/Reg/Max | 38/40.1/42.7 | [2]: Body temperatures measured by radio transmitters from 15 pikas in ambient temperatures -9.3 to 24°C ranged from 37.9-42.7°C (mean = 40.1)  [3]: Body temperatures of 12 pikas measured by rectal thermometer in ambient temperatures 2.0 to 24.4°C ranged from 39.3-41.0°C (mean = 40.1)  Body temperature of 1 pika measured by radio telemetry in ambient temperatures 10.0 to 27.6°C ranged from 39.1-41.0°C (mean = 40.4) |
| Flesh thermal conductivity (W/mC) | 0.4-2.8 | [4]: A collection of thermophysical property data on biological media, including a cold living hand (0.34 W/mC) and very warm living skin (2.8 W/mC)., and living skin with blood flow (1.58 – 7.0 W/mC). |
| O_2_ extraction efficiency (%) | 20% | [5]: Mammal O2 extraction efficiency is typically 20%. |
| Activity energy included in heat balance (%) | 80% | [5a]: Based on measurements of mammalian muscle efficiency being around 20% as measured in rats and mice. |
| Hair solar reflectivity  Dorsal/Ventral (%) | 57/45 | Our measurements of average reflectivity across 350-2500nm wavelengths on mid-dorsal fur samples from one summer pelt from the UW Zoological Museum. |
| Hair length (mm)  Dorsal/Ventral | Summer:  15/13  Winter:  22/19 | Our measurements on a summer pelt from Colorado from the UW Zoological museum: 14/12  [6]: Average hair lengths (n=3) from three pikas in summer pelage from Oregon: 15.3, 16.0, 16.9.  Winter pelage hair lengths assumed to be 50% longer based on [7]  July and August simulations used summer hair length; June and September simulations used an average of summer and winter hair length, on the assumption molt occurs during these months. |
| Fur depth (mm) | 50% of hair length | Our estimation (see sensitivity analyses) |
| Hair diameter (μm) | 31 | Our estimation |
| Fur density (per cm^2^) | 5000 | [8] & [9] Our estimation (see sensitivity analyses) based on values in the literature |

[1] Smith A.T. and Weston M.L. (1990) Ochotona princeps. American Society of Mammalogists. Mammalian Species No. 352.

[2] MacArthur RA and Wang LCH (1973) Physiology of thermoregulation in the pika, Ochotona princeps. *Can J Zool* 51: 11-16.

[3] Smith AT (1974) The distribution and dispersal of pikas: influences of behavior and climate. *Ecology* 55: 1368-1376.

[4] Chao BT, editor (1969) Advanced Heat Transfer. Urbana, IL: U Illinois Press. 459 pp.

[5] Lacombe A (2002) Effects of temperature on metabolism, ventilation, and oxygen extraction in the Southern Brown Bandicoot *Isoodon obesulus* (Marsupialia: Peramelidae). *Physiol Biochem Zool* 4: 405-411.

[5a]: Smith NP, Barclay CJ, Loiselle DS (2005) The efficiency of muscle contraction. *Progress in Biophysics & Molecular Biology* 88: 1-58.

[6] Johanna Varner unpublished data

[7] Howell, A.H. (1924) Revision of the American pikas. North American Fauna 47:1-57.

[8] Tregear, R.T. (1965) Hair density, wind speed, and heat loss in mammals. *J. Appl. Physiol*. 20(4), 796–801

[9] Davis, L.B., Jr., Birkebak, R. C. (1971) An analysis of energy transfer through fur, feathers, and other fibrous materials. Lexington, Ky.: Univ. Kentucky. Ph.D. diss., (as cited in Skuldt DJ, Beckman WA, Mitchell JW, Porter WP. 1975. Conduction and radiation in artificial fur. pp. 549-558 in Gates DM and RB Schmerl, eds., Perspectives of Biophysical Ecology. Ecological Studies Volume 12. Springer, Berlin.)

**Table S4.** Standard microclimate model inputs for Niche Mapper simulations of American pikas (*Ochotona princeps*) in Glacier

National Park, Montana, USA.

| **Parameter (units)** | **Value** | **Source** |
| --- | --- | --- |
| Substrate thermal conductivity (W/mK) | 1.75 | [1,2]: Estimated based on values from various rocky substrates obtained online. |
| Substrate density (kg/m^3^) | 2560 | [1,2]: Estimated based on values from various rocky substrates obtained online. |
| Substrate specific heat (J/kg-K) | 870 | [1,2]: Estimated based on values from various rocky substrates obtained online. |
| Substrate surface reflectivity | 25% | [1,2]: Estimated based on values from various rocky substrates obtained online. |
| Timing of daily minimum and maximum air temperatures and wind speeds (min/max) | Sunrise/1 hour after solar noon | Our estimation |
| Timing of daily minimum and maximum relative humidity (min/max) | 1 hour after solar noon / Sunrise | Our estimation |
| Percent cloud cover | 0-25% | Best fit to observed data |
| Percent shade | 0% | Assuming no trees in talus fields. |
| Daily wind speed range (m/s) | 0.1-4 | Best fit to observed data; pikas not active in wind speeds > 4 m/s. |
| Daily maximum relative humidity (%) | 100% | Our estimation |
| Animal height (cm) | 9 | Our estimation based on personal observation |

[1]Clauser, C. and E. Huenges (1995). Thermal Conductivity of Rocks and Minerals. Rock Physics and Phase Relations, A Handbook of Physical Constants. Hannover, Germany, Am. Geophysical Union**:** 105-126.

[2]Eppelbaum, L., I. Kutasov and A. Pilchin (2014). Thermal Properties of Rocks and Density of Fluids. Applied Geothermics, Springer Berlin Heidelberg**:** 99-149.
